# Gender and contextual variations in self-perceived cognitive competence

**DOI:** 10.3389/fpsyg.2022.919870

**Published:** 2022-11-03

**Authors:** Olivia Kuzyk, Alice Gendron, Luz Stella Lopez, William M. Bukowski

**Affiliations:** ^1^Department of Psychology, Concordia University, Montréal, QC, Canada; ^2^Department of Education, Universidad del Norte, Barranquilla, Colombia

**Keywords:** cognitive competence, gender, childhood, culture, socioeconomic factors

## Abstract

School performance and cognitive competence can be conceptualized as social and relational constructs. Thus, we expect their association to vary as a function of other socially-embedded variables which have proven meaningful in the academic domain. The present study takes a critical theory approach to assess gender-related and contextual variability in the association between peer-assessed school performance and self-perceived cognitive competence. The sample consisted of 719 preadolescents (M age = 9.5 years, range = 9 to 12.5 years) living in lower- and upper-middle-class neighborhoods in Montreal, Canada and Barranquilla, Columbia. Multigroup comparisons revealed that (a) peer-assessed school competence was more strongly associated with self-perceived cognitive competence for upper-middle-class than lower-middle-class participants from Barranquilla, whereas the opposite pattern was observed with Montreal participants, and (b) that the association between communal orientation and self-perceived cognitive competence was stronger for girls than for boys across the sample, especially in the upper-middle-class school in Montreal. These findings highlight the nuanced degree of gender differences in preadolescents’ perceived academic competence and emphasize the role of SES in shaping self-perceptions.

## Gender and contextual variations in self-perceived cognitive competence

The self is a dynamic construct that is shaped by experiences across the lifespan – especially in middle-childhood and adolescence (DuBois et al., 2000; Sebastian et al., 2008). Multiple proximal and distal factors influence the development of the self, including positive and negative aspects of individual experience (e.g., success and failure in achievement-related and social tasks), as well as other features of the school and peer environments (Bukowski and Raufelder, 2018). The present cross-cultural study emphasizes the intersection between gender-related features at the level of the person and the contexts where the children are situated. The study examines the associations between variations in self-perceived cognitive competence, school performance, and aspects of gender in a sample of preadolescent girls and boys from upper- and lower-middle-class families in Montreal, Canada, and Barranquilla, Colombia. The goal of the study was to examine (a) how preadolescents’ self-perceived cognitive competence is associated with school performance and with different aspects of gender and (b) how these associations vary as a function of cross-cultural context (i.e., place) and socioeconomic status (SES).

Self-perceived competence is defined as an individual’s judgment of their own abilities, functioning, and well-being (Harter, 1996). Research on the self is typically guided by three premises (Harter, 2012); (1) that self-perceptions are only moderately associated with actual experiences, (2) that self-perceived competence can be affected by other person-related variables that can either increase or decrease a person’s negative or positive self-views, and (3) that person-level and group-level variables can moderate the association between measures of functioning and self-perceptions. We used a broad multilevel perspective in our application of these premises to the study of the effects of gender and self-perceptions on cognitive competence. Our approach is characterized by two central ideas. The first is that we recognize that gender is a complex and multifaceted construct whose features need to be studied together to obtain a fuller view of how the defining aspects of gender work in concert to affect outcomes. Second, we maintain that gender is a social construct whose defining characteristics and given meanings are likely to vary across social and cultural contexts (World Health Organization [WHO], 2017). Incorporating these points related to the self and gender into our framework present some theoretical challenges, which are addressed in the following sections.

### Gender theory

Our approach to these issues is inspired by critical theory (Bohman, 2021), and more specifically, by three fundamental claims from critical gender theory (Jule, 2014). The first claim is that simple comparisons between females and males provide only a very narrow assessment of the vast array of features and effects that constitute gender. In this study, we go beyond a simple binary comparison by including measures fashioned after the femininity and masculinity measures of the Bem Sex Role Inventory (BSRI) in order to capture gender-role traits that covary with cisgender measures of masculinity and femininity (Bem and Lewis, 1975). A second claim of critical gender theory is that to understand the dynamics of gender, one needs to assess how the facets of gender interface with actual experiences and social institutions.

In this study, we assessed how gender-related traits are associated with school performance. Instead of seeing school performance solely as a form of individual achievement, we also conceptualize it as a relational or participatory experience which may benefit from one’s capacity to connect with the shared goals of the institutional environment. We see the gendered dimension of communal orientation as a trait that promotes effective functioning in academic tasks. We examined these factors by assessing how aspects of gender were related directly to self-perceived cognitive competence and how they moderated the association between school performance and self-perceived cognitive competence. The third claim is that gender is a social construct whose features and meanings vary across contexts. We assessed contextual variance by examining the effects of two broad contextual factors: the socioeconomic status of the children’s school/neighborhood and place (i.e., whether participants were from Montreal. Canada, or Barranquilla, Colombia). We chose to study preadolescents from two cultural contexts that were likely to differ in their normative social relationship to gender and its multiple facets, as well as display differences in the gendered experiences they present to children in their respective settings when comparing lower- and upper-middle-class school environments. This decision is based on prior findings with classroom samples that support variance in gender identity as a function of SES (Bukowski et al., 2019, 2021). Our assessment of contextual factors focused on between-group differences in the degree to which associations between measures of gender (i.e., cisgender and gender roles) were associated with measures of self-perceived cognitive competence, and assessed whether gender moderated the association between school performance and self-perceived cognitive competence.

### Self-perceived cognitive competence in an academic setting

Self-perceptions of academic competence are an understudied domain of research in relation to the self-concept. They are important because they affect subsequent goals in school tasks (Bong and Skaalvik, 2003; O’Mara et al., 2006). Children who endorse positive views of their cognitive competence have been shown to make more efforts to perform academically (Guay et al., 2003). Research also supports that academic attainment influences children’s self-concept during a developmental period where academic self-perceptions are sensitive to experiences of success and failure (Skaalvik and Valås, 1999). As such, a bi-directional model (Marsh and Martin, 2011; Brunner et al., 2013) offers a more complete understanding of the reciprocal contributions between academic self-perceptions and performance.

There is a historical trend of gender differences in school performance throughout elementary school and into adolescence (e.g., Brophy, 1985; Alexander et al., 1997; Dwyer and Johnson, 1997; Neuburger et al., 2012; Kingdon et al., 2017). Current evidence shows that the difference between girls and boys is relatively small (Voyer and Voyer, 2014) except perhaps in academic subjects that rely heavily on language skills (Reilly et al. (2019). There is some long-standing evidence that girls perceive themselves more positively in stereotypically feminine areas (i.e., reading and writing), but judge themselves more harshly on stereotypically masculine subjects (i.e., math and science) (Ruble et al., 1993). Moreover, girls evaluate themselves more negatively on measures of general self-worth (Kling et al., 1999) and report higher levels of school-related worry compared to boys (Silverman et al., 1995).

Explanations for this discrepancy may, in part, centre on differences on how boys and girls develop a sense of cognitive competence. Generally, researchers highlight that boys show higher scores on measures of self-worth compared to girls (e.g., Chubb et al., 1997; Quatman and Watson, 2001; Birndorf et al., 2005). This may be reflective of how boys and girls approach academics and manage evaluative feedback. That is, girls may regard these situations as opportunities to learn about their abilities and thus, may be more likely to internalize feedback (Roberts, 1991). These tendencies may motivate girls to do well, and also lead them correspondingly to experience more distress when they encounter failure or difficult feedback. Boys, conversely, are more competitive and may approach academics with more self-confidence and deny the evaluative feedback that is provided (Roberts, 1991). A self-confident approach may buffer the effects of failure or poor performance because it may lead boys to view feedback as less informative. This model signals potential variation in how children perceive their cognitive competence and perform in school based on the extent to which they ascribe to masculine and feminine traits.

Therefore, one can speculate that the processes of academic achievement can be conceptualized in relation to forms of functioning that are differentially associated with feminine and masculine gender roles. The items assigned to the feminine and masculine scale of the Bem Sex Role Inventory can be conceived of as fitting the well-established dimensions of communion and agency that are known to be gendered aspects of functioning (Abele and Wojciszke, 2007; Abele et al., 2016). Whereas the personal features of instrumentality and assertiveness associated with the masculine gender role (Bem, 1974) support a view of academic competence as a form of individual achievement that results from personal action, the personal feature of communion associated with the feminine gender role may support a view of competent school functioning as a collective activity that requires a commitment to group processes, which derive from group-sanctioned forms of knowledge. Hence, one could expect communion to promote self-perceived academic competence to the degree that functioning in school rests on participation in communal activities. According to this perspective, adhering to gender roles and perceiving oneself as cognitively competent may be overlapping forms of self-perception. As a result, boys who see themselves as being assertive and instrumentally competent may also see themselves as academically competent, as this form of competence is an expression of their personal assertiveness and instrumental skills. Similarly, girls who see themselves as communal may also see themselves as academically competent as this form of competence is an expression of their capacity to function in a domain that requires communal skills. This perspective is supported by evidence that adolescents perceive schools to be more feminine than masculine (Heyder and Kessels, 2013). Based on this reasoning, one can hypothesize that (a) gender roles may be univariate correlates of measures of self-perceived cognitive competence and (b) they may function as moderators that strengthen the association between achievement and self-perceived cognitive competence. Each of these hypotheses will be examined in our analyses.

### Self in context

Socioeconomic status and culture are likely to affect variations in the self-concept during preadolescence. Children are situated within rich networks of influence, and thus it is unsurprising that these contexts impact their self-worth and academic achievement. Children belonging to high-SES families report higher levels of self-worth compared to those from low-SES backgrounds (Rhodes et al., 2004). However, this relationship is dependent on the importance placed on academic achievement. Campbell et al. (2002) have reported that this pattern has been observed as a result of a stronger emphasis placed on academic achievement in high-SES families.

Similarly, Santo et al. (2013) found that cognitive competence was more strongly related to self-worth in a sample of early adolescents from a low-SES background, whereas social competence was more closely linked to self-worth among children in high-SES groups. The authors posited that these patterns reflect differences in culturally-determined indicators of self-worth. For low-SES peer groups, pursuing an education may be a strong indicator of success, whereas achieving or maintaining social status may be more important for higher-SES families. Divergent patterns were also observed for adolescents from individualistic cultures relative to collectivistic cultures. More specifically, the association between perceived cognitive competence and self-worth was weaker for those from a collectivistic society compared to those from an individualistic one. Cognitive competence may be more closely aligned with individualistic values and thus regarded as less important to collectivistic groups (Santo et al., 2013). There is no doubt that differences in how early adolescents perceive their cognitive competence and general worth are complex and salient across cultural groups.

### The current study

Broadly, the focus of the present study was to examine contextual variations in how young adolescents’ self-perceived cognitive competence is associated with their academic achievement. Here, gender is the primary contextual variable of interest, in that the current research builds on historical trends of gender differences in scholastic performance and provides objective indices of how they relate to cognitive competence. We conceptualize gender as a multidimensional construct. The current research examined the extent to which self-assessed cognitive competence and peer-assessed academic performance varies as a function of both masculine and feminine features of gender. It was hypothesized that children who identify more strongly with feminine features will demonstrate a stronger association between perceived cognitive competence and academic achievement.

Moreover, in light of research emphasizing the importance and complexity of the cultural context, we aimed to examine how this relationship changes across interactions of SES and cultural groups. We proposed that for children who identify with feminine traits, the relationship between their self-perceptions of cognitive competence and academic achievement will be strongest in the low-SES individualistic group. Second, we hypothesize a significant association between cognitive competence and academic achievement for children who reported more feminine traits in the low-SES collectivistic group.

## Methods

### Participants

The sample consisted of 719 (M age = 10.70 years, SD = 1.20) fourth-, fifth- and sixth-grade girls (*N* = 380) and boys (*N* = 339) in mixed-sex schools located in lower-middle- and upper-middle-class neighborhoods in Montreal, Canada (*N* = 302) and Barranquilla, Colombia (*N* = 417). The proportion of boys and girls, and of upper- and lower-middle-class participants, was roughly the same in each country. Socioeconomic status was operationally defined with different criteria for the two places. In Colombia, this designation was based on an index of neighborhood SES known as *estrato* that is assigned by the Colombian government based on the quality of housing and services in the neighborhood (Rueda-García, 2003). Scores range from 1 to 6, with higher scores indicating greater affluence. The mean estrato score for the children from lower-middle-SES schools was 2.52, (SD = 0.70) indicating that the participants at the low-SES schools were indeed within the lower socioeconomic strata. Although individual estrato ratings were not obtained from the high-SES school sampled in Barranquilla, school officials indicated that children who attended this school typically fell into the highest estrato category (6). The data were collected in 2002.

SES for the Montreal children was based on the average family income of children in their school. Parents completed a questionnaire in which they selected the income level (from 10 choices ranging from below $15,000 to over $95,000) that was closest to that of each adult member of the household in the last year. A total income score was calculated by adding the income of each family member. There were large between-school differences: one school had a mean family income of $36,027 CND, a second school had a mean of $68,400 and the third school had a mean of $79,194. The first school was designated as lower-middle class and the second two schools as upper-middle class. Based on information from the 2001 Canadian census (the census conducted closest to the time of the data collection), the mean family income of participants from the first school was considerably lower than the provincial average of $59,296, whereas the mean family income of participants in the latter two schools was above the provincial average (Statistics Canada, 2002). In the Barranquilla part of the sample, there were 149 participants from the two schools in lower- middle-class neighborhoods and 268 participants from the one school whose students came from upper-middle-class neighborhoods. In the Montréal part of the sample, there were 149 participants from the one school in a lower- middle-class neighborhoods and 268 participants from the two schools in upper-middle-class neighborhoods.

### Procedure

A multi-stage recruitment process was used in each city. In Montreal, permission was first obtained from the relevant school board, and then from school principals. Active consent was required from parents of potential participants. In Barranquilla, the parents of the potential participants were informed by the school principal of the purposes and procedures of the study. They were also informed that participation in the study was voluntary. Parents could ask for their child not to be included in the study. In this region of Colombia, school principals often act *in loco parentis.* Their rights as participants were explained to them prior to the beginning of the data collection. Each participating child provided assent to be in the study. Using these recruitment procedures, a participation rate of over 85% was obtained in Montreal and of over 90% in Barranquilla.

The children completed a questionnaire at their desks in their classrooms in a group administration. The Colombian participants completed a version of the questionnaire that had been translated into Spanish by translators working in the areas of education and psychology. This adaptation was also backtranslated into English by a separate group of translators to ensure that the meaning of items was retained in the process.

### Measures

The participants completed three measures: (a) a peer assessment measure of school performance, (b) an altered version of Harter’s (1982) Perceived Competence Scale for Children, and (c) an abbreviated version of the Bem Sex Role Inventory Bem (1974). The participants completed these inventories *via* a paper-and-pencil format at their desks in class. At least three members of the project team were in each classroom to make sure the participants understood the instructions and to answer any questions about how to complete the measures.

### Peer assessment measure

Peer assessment procedures are used to assess how children are perceived by their peers. These procedures are known to provide valid and reliable measures of children’s competence and effective functioning (Bukowski et al., 2012). In a peer assessment procedure, participants are shown a list of items that describe forms of functioning and are asked to indicate which of their participating classmates fit each description. In this study, two items were used to assess school performance. They were “Someone who is smart and does well in school” (“Es intelligente y tiene un buen rendimiento academico en le escuela”) and “Someone who always knows the right answers in school” (“Siempre sabe la respuesta correcta en la escuela”). Two scores were calculated for each participant on each item. These values correspond to the number of times the child was nominated for the item by same- and other-gender peers. Each score for each item was adjusted for possible biases that may result from variations in group size (see Velásquez et al., 2013). Separate corrections were made for the same-gender and other-gender measures. In this study, only the same-gender measures were used. A school performance score was computed for each participant by adding the two class-size-adjusted same-gender scores together. When assessed using Cronbach’s alpha, the reliability of this aggregated score was observed to be 0.92. The use of a peer assessment measure is advantageous as it provides a common measurement procedure across the schools and contexts included in the study. Other forms of measurement, such as school grades, can be problematic due to variations in the procedures used in different schools and places. The mean and standard deviation for this measure are 1.22 and 1.88.

### Bem sex role inventory

The participants rated ten words taken from Bem’s (1974) BSRI. Two of these words were “feminine” and “masculine;” the other eight words were chosen based on two criteria. First, we chose words for which there was strong empirical evidence of their alignment with the femininity and masculinity dimensions used in Bem’s (1974) initial studies. Second, the words had to be relevant to the preadolescent participants in the study. The four words were taken from the femininity scale were “Affectionate,” “Sympathetic,” “Understanding,” and “Sensitive to the Needs of Others.” This set of items was seen as representative of communal orientation. The four words taken from the masculinity dimension were “Independent,” “Athletic,” “Leader” and “Forceful.” They were interpreted as representing instrumentality/assertiveness. Using a five-point scale in which a “1” represented “Not like me at all” (“No me describe”) and a “5” equated to “Just like me” (“Me describe”), each participant rated each word according to whether it provided a true description of the self. The scores on the items for each measure were initially analyzed with a principal components factor analysis. The observed factor loadings were used to create a communal orientation score and an instrumentality/assertiveness score for each participant. To create these scores, the items were weighted by the observed factor loadings from the PCA. The internal consistency of these scales, assessed with omega, was 0.77 and 0.82 for the instrumentality/assertiveness and communal orientation scales, respectively. The mean and standard deviation for the instrumentality/assertiveness measure are 3.69 and 1.00; the mean and standard deviation for the communal orientation measure are 3.93 and 0.97.

### Perceived competence scale for children

Self-perceived cognitive competence was measured using selected items from Harter’s (1982) Perceived Competence Scale for Children. A set of seven items adapted from Harter’s original scale were used to assess positive views of cognitive competence. Consistent with the rating scale concerns raised by Yeager and Krosnick (2011), the items were written to fit a simple five-point scale in which 1 meant “never true” and 5 meant “always true.” The preadolescents were instructed to read each description and indicate how well each one fit their self-view. The items were “I feel that I am very good at my school,” “I feel like I am just as smart as other kids my age,” “I like school because I do well in school,” “I am pretty slow in finishing my schoolwork,” “I often forget what I learn,” “I wish it were easier to understand what I read,” and “I have trouble figuring out the answers in school.” The last four items were reversed self-perceived competence items. As with the procedures used with the BSRI items, the scores on these items were initially analyzed with a principal components factor analysis. The observed factor loadings were used to create a self-perceived competence score for each participant. To create this score, the items were weighted by the observed factor loading from the PCA. The internal consistency of this scale, assessed with omega, was observed to be 0.76. The mean and standard deviation for this measure are 3.65 and 0.79. (The means (and standard deviations) for all the person-level variables are shown in Table 1 for the categorical combinations of cisgender, SES, and place.)

**TABLE 1:** Means (standard deviations) for person-level variables for groups defined by participant gender, SES, and place.

Group	Self-perceived cognitive competence	Peer-assessed school performance	Communal orientation	Instrumentality/ Assertiveness
Boys, Barranquilla, Lower Middle Class	3.40 (0.72)	0.84 (1.41)	3.92 (0.88)	3.92 (0.94)
Girls, Barranquilla, Lower Middle Class	3.53 (0.79)	1.01 (1.49)	4.11 (0.81)	4.00 (0.84)
Boys, Montréal, Lower Middle Class	3.75 (0.91)	1.36 (1.69)	3.91 (0.84)	3.99 (0.93)
Girls, Montréal, Lower Middle Class	3.96 (0.76)	1.45 (1.70)	4.34 (0.52)	3.66 (0.72)
Boys, Barranquilla, Upper Middle Class	3.62 (0.75)	1.22 (1.99)	3.63 (1.18)	3.60 (1.20)
Girls, Barranquilla, Upper Middle Class	3.70 (0.79)	1.09 (1.86)	3.93 (1.21)	3.47 (1.04)
Boys, Montréal, Upper Middle Class	3.51 (0.77)	1.27 (1.72)	3.77 (0.87)	3.76 (1.03)
Girls, Montréal, Upper Middle Class	3.69 (0.78)	1.30 (1.65)	3.96 (0.75)	3.54 (0.97)

Other variables included in the analyses were place, coded as −1 for Montreal and 1 for Barranquilla, cisgender (i.e., the gender assigned to the child at birth) coded as −1 for boys and 1 for girls, and SES coded as −1 for lower-middle-class and 1 for upper-middle-class.

## Results

Analyses were conducted with Mplus (Muthén and Muthén, 2015). A two-phase procedure followed. In the first phase, person-level variables were used as predictors of the outcome measure (i.e., the measure of self-perceived cognitive competence). In the second phase, mulitigroup comparisons were performed to assess whether any of the associations observed in the first phase differed as a function of place (i.e., Barranquilla and Montreal), SES, and the intersection between place and SES.

In the first phase, eleven variables were used as predictors of the dependent variable (i.e., self-perceived cognitive competence). These predictors were used to capture the univariate and the interactive effects of the peer-assessed measure of academic performance and the three gender measures. The eleven predictors were: (a) the peer-assessed measure of school performance, (b) the participant’s cisgender, (c) the measure of instrumentality/assertiveness, (d) the measure of communal orientation, (e) the two-way interaction between the peer-assessed measure of school performance and the cisgender measure, (f) the two-way interaction between peer-assessed school performance and communal orientation, (g) the two-way interaction between peer-assessed school performance and the measure of instrumentality/assertiveness, (h) the two-way interaction between the cisgender measure and communal orientation, (i) the two-way interaction between the cisgender measure and the measure of instrumentality/assertiveness, (j) the three-way interaction between the cisgender measure, peer-assessed school performance, and communal orientation, and (k) the three-way interaction between the cisgender measure, peer-assessed school performance, and instrumentality/assertiveness. The statistically significant findings are reported in Table 2.

**TABLE 2:** Person-related predictors of the self-perceived cognitive competence score.

Level 1 variable	Standardized coefficients (standard errors)	*t* score (P-value)
Peer Measure	0.36 (0.03)	10.90 (0.001)
Cisgender	0.08 (0.03)	2.33 (0.02)
Communal Orientation	0.12 (0.04)	3.38 (0.001)
Peer Measure by Cisgender	−0.10 (0.04)	−2.87 (0.005)
Cisgender by Communal Orientation	0.07 (0.04)	2.05 (*p* < 0.05)

Initial analyses revealed statistically significant coefficients for five of the predictors, specifically (a) the peer-assessed school competence measure (standardized coefficient = 0.36, standard error = 0.03 *t* = 10.90, *p* < 0.001), (b) cisgender (standardized coefficient = 0.08, standard error = 0.04 *t* = 2.33, *p* < 0.02), (c) communal orientation (standardized coefficient = 0.12, standard error = 0.04, *t* = 3.38, *p* < 0.001), (d) the two-way interaction between the peer-assessed school performance measure and cisgender (standardized coefficient = –0.10, standard error = 0.035, *t* = –2.87, *p* < 0.005), and (e) the two-way interaction between communal orientation and cisgender (standardized coefficient = 0.071, standard error = 0.039, *t* = 2.44, *p* < 0.15). A clarification of the two-way interaction between the peer-assessed school performance measure and cisgender indicated that the association between self-perceived cognitive competence and peer-assessed school performance was stronger for boys (coefficient = 0.43) than girls (coefficient = 0.27). A clarification of the two-way interaction between communal orientation and cisgender indicated that the association between self-perceived cognitive competence and communal orientation was stronger for girls (coefficient = 0.19) than for boys (coefficient = 0.04).

Multigroup comparisons, conducted with Mplus, were then performed to assess whether these associations differed (a) for the participants from the two places, (b) for the participants from the lower-middle-class and upper-middle-class schools, and (c) for the participants from the four groups defined by a combination of place and SES (i.e., lower-middle-class participants from Barranquilla, lower-middle-class participants from Montreal, upper-middle-class participants from Barranquilla, and upper-middle-class participants from Montreal). Each multigroup comparison consisted of a two-step process (see Wang and Wang, 2019). In the first step, equality constraints were used to set the coefficients for a particular association to be equal across groups (e.g., the upper-middle-class and the lower-middle-class participants). If the coefficients for these groups were equal to each other, then setting them to be equal would not affect the overall fit of the model. If the coefficients were not equal to each other, then setting them to be equal would have an adverse effect of model fit. This negative effect of model fit would be manifested in an increase in the Chi-square value. In the second step of this comparative procedure, a chi-square difference test was used to assess the statistical significance of the change in the chi-square value.

Comparisons of the coefficients observed with the participants from the two places revealed no statistically significant differences. Comparisons that assessed differences between the participants from lower-middle-class and upper-middle-class schools revealed only one statistically significant difference. Specifically, the two-way interaction between cisgender and communal orientation was observed to be weaker and statistically non-significant with the participants from the lower-middle-class schools (standardized coefficient = –0.03, standard error = 0.058, *t* = −0.53, *p* > 0.5), whereas it was statistically significant with the participants from the upper-middle-class schools (standardized coefficient = 0.13, standard error = 0.044, *t* = 2.88, *p* < 0.005). The positive coefficient observed with this two-way interaction for the participants from the upper-middle-class schools indicates that the effect of a communal orientation was stronger for girls than for boys.

Multigroup comparisons conducted with the four groups defined by a combination of place and SES revealed three between-group differences. First, the measure of peer-assessed school competence was observed to be more strongly associated with the outcome measure for the upper-middle-class participants from Barranquilla (standardized coefficient = 0.41) than for the lower-middle-class participants from Barranquilla (standardized coefficient = 0.26). The corresponding values for the upper-middle-class and lower-middle-class participants from Montreal were 0.28 and 0.36, respectively. These coefficients did not differ from each other. All of these coefficients were statistically significant. It is important to note that the differences between the upper-middle-class participants and lower-middle-class participants showed a different pattern in Montreal (lower-middle class was higher than upper-middle class) than in Barranquilla (lower-middle class was lower than upper-middle class).

A second difference was observed with the association between communal orientation and self-perceived academic competence. This association was observed to be more stronger for the lower-middle-class participants from Montreal (standardized coefficient = 0.19, standard error = 0.082, *t* = 2.25, *p* < 0.02) than for the lower-middle-class participants from Barranquilla (standardized coefficient = −0.024, standard error = 0.082, *t* = 0.29, *p* < 0.75). The corresponding values for the upper-middle-class participants from Montreal and Barranquilla were 0.07 (standard error = 0.075, *t* = 0.96, *p* > 0.3) and 0.14 (standard error = 0.055, *t* = 2.63, *p* < 0.009). Again, a different pattern of findings was observed in Montreal (lower-middle class was higher than upper-middle class) than in Barranquilla (lower-middle class was lower than upper-middle class).

The third set of differences was observed with the association between two-way interaction between cisgender and communal orientation and the measure of self-perceived academic competence. The coefficients for the association between this interaction score and the outcome were observed to be positive and statistically significant with the participants from upper-middle-class schools in Montreal (standardized coefficient = 0.14, standard error = 0.07, *t* = 1.98, *p* < 0.05) and Barranquilla (standardized coefficient = 0.12, standard error = 0.055, *t* = 2.15, *p* < 0.03) and negative and statistically non-significant with the participants from lower-middle-class schools in Montreal (standardized coefficient = –0.09, standard error = 0.082, *t* = −1.29, *p* > 0.3) and Barranquilla (standardized coefficient = –0.022, standard error = 0.082, *t* = –0.26, *p* > 0.7). Group comparisons indicated that the coefficients for the participants from the upper-middle-class school differed from the coefficient observed with the participants from the lower-middle-class schools in Montreal. The positive value of the coefficients observed with the participants from the upper-middle-class school indicates that for these participants, the association between communal orientation and the outcome measure (i.e., the measure of self-perceived cognitive competence) is stronger for girls than for boys.

## Discussion

Two key findings were revealed. The first is that the measures of gender roles are associated with self-perceived cognitive competence as univariate predictors and as moderators. As importantly, our findings were varied as a function of place and SES. These findings point to the complex pattern of the factors associated with self-perceived cognitive competence and its association with specific components of gender. The findings confirm two basic features of the study’s conceptual frame. Specifically, the findings show that the associations observed with gender-related variables will vary as a function of contextual factors – especially intersection between culture and SES. The findings also show that adherence to gender roles is associated with self-perceived cognitive competence in a direct manner and as a moderator of experience. This evidence of the importance of gender role adherence was, however, observed only with the dimension of communal orientation and only in particular contexts.

A primary finding from the study is the observation that the association between peer-assessed school performance and self-perceived cognitive competence is moderated by the cisgender measure, and that this interaction is moderated by an interaction between place and SES and by the cisgender measure. Peer-assessed school competence was observed to be more strongly associated with the outcome measure for the upper-middle-class participants from Barranquilla than for the lower-middle-class participants from Barranquilla. The opposite pattern was observed with the Montreal participants; albeit to a smaller and statistically non-significant degree. The moderating effect of the cisgender measure indicated that the association between peer-assessed school performance and self-perceived cognitive competence was weaker for girls than for boys. Consistent with prior findings, self-perceptions of cognitive competence appear to be less dependent on actual experience for boys than for girls. These findings confirm our prior results, observed with a different sample, that gender differences may be stronger for upper-middle-class children in the Colombian context (Santo et al., 2013). They also provide an explanation for Van Houtte’s (2004) observation of stronger achievement levels among boys than girls.

The second important result pattern also points to a difference between girls and boys. This two-way interaction indicates that the association between communal orientation and self-perceived cognitive competence was stronger for girls than for boys. This finding provides partial support for our reasoning that the gender-role measure may overlap with the self-perceived competence measure. To a small degree, girls who see themselves as communally oriented also see themselves and being competent in cognitive tasks. This pattern was further moderated by SES and place, and was seen only among the participants from the upper-middle-class school in Montreal. Hence, this shows that the effect of gender varies as a function of culture and SES. In this way, these findings provide partial support for our speculation that gender roles are intertwined with perceptions of cognitive competence. This evidence was, however, limited in two ways. First, it was observed only with the measure communal orientation. Second the effect of communal orientation was observed only for girls from upper-middle-class neighborhoods in the two places. These findings reveal a high level of specificity in gender-related findings. Together, these findings emphasize the importance of gender in models of self-perceived academic competence.

Our analyses revealed three statistically significant univariate findings and two statistically significant two-way findings at the level of the person. Positive associations were observed between self-perceived cognitive competence and (a) peer-assessed school performance, (b) the cisgender measure (i.e., girls showed stronger judgments of their cognitive abilities than boys) and (c) communal orientation. Additionally, statistical analyses involving two-way interactions across these measures (i.e., cisgender and school performance; cisgender and communal orientation) revealed that academic achievement was more predictive of boys’ self-judgments of their cognitive competence as compared to girls, whereas communal orientation was more predictive of self-perceived cognitive competence for girls.

The group comparisons show that the meaning of gender around scholastic achievement and self-assessed cognitive competence is contextually dependent, particularly across SES groups. That is, a two-way interaction between cisgender and communal orientation was predictive of cognitive competence among children attending upper-middle-class schools, but not lower-middle-class schools. Notably, this effect appeared stronger for girls relative to boys. Between-group comparisons further highlighted the complexities of contextual variations, in that communal orientation was related to self-assessed cognitive competence in lower-middle-class schools in Montreal, but not Barranquilla. Additionally, the interaction between cisgender and communal orientation was predictive of the outcome in upper-middle class schools in both Montreal and Barranquilla. Analyses also revealed that this effect was stronger for girls than it was for boys. Perhaps the most important finding from the study is the observation that the self-perceptions of cognitive competence among girls and boys from lower SES neighborhoods in Barranquilla appear to be unaffected gender roles. This finding is important as it supports the basic premise of the study that the significance of gender varies across cultural contexts. Although an exact interpretation of this pattern of findings is elusive, at the very least they indicate that the meaning of the measures of gender used in our analyses are different for the low SES participants from Barranquilla. It may be that the concepts themselves (i.e., a communal orientation and assertiveness/instrumentality) are not as “gendered” for the low SES participants from Barranquilla. A further exploration of these findings may benefit from an assessment of how these measures are associated with gender-related constructs such as gender typicality and felt pressure to conform (see Egan and Perry, 2001) to conform and whether these associations vary as a function of SES and culture. Together, these findings indicate that the SES composition of classrooms across geographic location shape the gender norms around academic achievement and cognitive competence.

Researchers have already suggested that hegemonic gender norms are evoked and sanctioned depending on the social context in which they occur (Ridgeway and Correll, 2004; Morris, 2011; Hsin, 2018). The school context is therefore a major channel for how these gender norms are expressed and actualized in young adolescents’ achievement outcomes (Hsin, 2018). Ethnographic studies demonstrate that divergent achievement patterns for boys and girls evolve from cultures of masculinity that minimize the importance of boys performing well academically (e.g., United Kingdom: Mac an Ghaill, 1994; Australia: Martino, 1999; United States: Pascoe, 2007). Certain academic disciplines and study behaviors are regarded as “feminine,” which has been shown to negatively affect boys’ motivation toward school (Pajares and Valiante, 2001; Bhanot and Jovanovic, 2005). In fact, traits linked to femininity as well as those that are consistent with studious attitudes (e.g., being tidy, cooperative and passive) may even be advantageous for girls (Jones and Myhill, 2004; Beaman et al., 2006). Our findings support this view. Other research has shown that boys’ peer groups in secondary school have tendencies to engage in less studious behaviors compared with girls, which notably accounts for the lower academic performance observed in boys (Van Houtte, 2004). Our findings fit well within this body of work. They highlight that the extent to which young adolescents, particularly girls, who identify with features of communal orientation also hold self-perceptions of their cognitive competence. A communal orientation may be largely consistent with the studious behaviors that have been identified in previous studies.

Furthermore, research has supported that gender differences in academics are strongly impacted by the SES composition of schools (Legewie and DiPrete, 2014). Legewie and DiPrete (2014) reported that high-SES classrooms promote girls’ academic achievement because they are not gendered as “feminine” in terms of interests and pursuits. Interestingly, they also encourage boys’ educational outcomes by influencing their choice in the science, technology, engineering and mathematics (STEM) fields. Qualitative studies further demonstrate how hegemonic gender expectations are promoted through school environments—particularly by emphasizing engagement in sports culture over academics for boys (Morris, 2008). Participating in sports reflects an expression of hegemonic masculinity by demonstrating toughness and physical strength (Morris, 2008). Taken together, schools with a high-SES compositions do not regard academic achievement as a feminine pursuit, but more subtly enforce gendered behavior and interests for boys through the promotion of STEM trajectories and sports engagement.

The present set of findings are also consistent with this view but may reflect differences in the extent to which gender expectations in academic achievement are actualized across the developmental trajectory. We observed that the gender differences on self-competence were strongest in upper-middle-class schools in Montreal and Barranquilla. This may signal to more salient gender norms around academics for girls in upper-middle-class SES compositions. Similarly, it is also possible that there is less emphasis on STEM trajectories and sports engagement for boys in early adolescence, and thus less opportunity to shape self-perceived cognitive competence. Research in the field of self-efficacy (i.e., individuals’ judgments around their ability to engage in behaviors that are required to achieve a desired objective) demonstrate gender differences emerging in early adolescence which increase over development (Huang, 2013). As such, we would expect divergent patterns to emerge as boys become exposed to more specific pressures for gender conformity as they progress in the school system and make choices about their future vocation. Specific academic courses were found to be important moderators of self-efficacy, in that previous work identified boys as having higher self-efficacy scores in mathematics and computer sciences, whereas girls showed elevations in language arts and small advantages on general academic self-efficacy (Van Houtte, 2004). Therefore, it would be of benefit for researchers to use statistical network analyses to examine how features of gender and cognitive competence vary as a function of academic courses, in addition to SES school composition and place variables.

Some limitations should be noted. First, the use of a cross-sectional design prevented causal interpretations. Follow-up studies using longitudinal designs are needed. Second, although multiple measure of gender were used, one can imagine that including more measures of gender identity would add diversity to the findings. Third, the data were collected 20 years ago. Given that some aspects of gender identity may have changed in the intervening years (Donnelly and Twenge, 2017), a replication study using more recent data is needed. Fourth, the study relies to a great extent on self-report measures. Aspects of gender and the dimensions of the self-concept are typically with self-report procedures. The use of peer reports might add to the currently available measures of gender. Fifth, SES is a multilevel concept (Bukowski et al., 2020). Although it is often measured as a feature of an individual or a family, SES was used here ad a measure of the school context. A more complex approach to SES that included measure at the level of the group and the individual would expand our understanding of how SES intersects with gender and self perceptions of competence. Sixth, a richer conceptualization of gender is needed to understand the degree to which gender identity should be conceived of as a trait or as a conscious form of self-perception related to one’s gender.

In conclusion, the present set of findings builds upon existing research to provide further insight into gender-related variations in self and academic achievement in early adolescence and across socio-geographical contexts. Our work highlights the specificity of gender differences in self-perceived cognitive competence in upper-SES compositions in schools, and thus ascribes meaning to features of gender that are dependent on gender expectations for scholastic achievement. While this study helps explain contextual variations in how young adolescents’ self-perceptions of their cognitive competence are associated with their academic achievement, further research is required to disentangle course-specific nuances in order to reduce gender gaps and promote equality in academic achievement.

## Data availability statement

The raw data supporting the conclusions of this article will be made available by the authors, without undue reservation.

## Ethics statement

The studies involving human participants were reviewed and approved by the Concordia University Human Research Ethics Committee approved the study and it conforms to the recognized standards by the Declaration of Helsinki. Written informed consent to participate in this study was provided by the participants’ legal guardian/next of kin.

## Author contributions

All authors listed have made a substantial, direct, and intellectual contribution to the work, and approved it for publication.
